# Evaluation of Chromogenic Culture Media for Rapid Identification of Gram-Positive Bacteria Causing Mastitis

**DOI:** 10.3389/fvets.2021.662201

**Published:** 2021-04-30

**Authors:** Breno Luis Nery Garcia, Carlos Eduardo Fidelis, Gustavo Freu, Brunna de Mattos Granja, Marcos Veiga dos Santos

**Affiliations:** Qualileite Milk Quality Laboratory, Department of Animal Nutrition and Production, School of Veterinary Medicine and Animal Sciences, University of São Paulo, São Paulo, Brazil

**Keywords:** chromogenic media, microorganism identification, mastitis, milk quality, on-farm culture

## Abstract

The present study aimed to evaluate the diagnostic performance specificity (Sp), sensitivity (Se), positive predictive value (PPV), negative predictive value (NPV), and accuracy (Acc) of two chromogenic culture media for rapid identification of Gram-positive bacteria causing subclinical mastitis (SCM) in dairy cows. For this, the performance of chromogenic culture media Gram-positive (GP) and *Staphylococcus* (Staph) (CHROMagar ™, Paris—France) was evaluated in milk samples collected from: (1) lactating cows with SCM (*n* = 504), and (2) cows in the post-partum period (PP) (7 ± 3 days post-partum; *n* = 536). Rapid identification of Gram-positive bacteria in chromogenic media was performed by visual inspection of colony colors after 24 h of incubation at 37°C. Bacterial identification by MALDI-TOF mass spectrometry was considered the reference methodology for calculating: Acc, Se, Sp, PPV, NPV, and Cohen's *Kappa* coefficient of agreement (*k*). The chromogenic media GP showed high Acc for *Strep. agalactiae/dysgalactiae* identification in both samples of SCM (Se: 89.1%; Sp: 96.3% and Acc: 95.6%) and of cows in PP (Se: 100%; Sp: 99.0% and Acc: 99.1%). Similar results were observed for *Strep. uberis/Enterococcus* spp. identification (Se: 90.5%; Sp: 92.5% and Acc: 92.3%) in SCM samples and Se: 100%; Sp: 99.6% and Acc: 99.6% in samples of PP cows using the GP media. However, the GP chromogenic media showed low Se (25.0% in SCM samples and 50.0% in samples of cows in PP) for *Staph. aureus* identification, despite Sp and Acc were high (Sp: 98.3% and Acc: 95.4% in SCM and Sp samples: 99.4% and Acc: 98.9% in PP cow samples). Staph culture media showed high Acc for *Staph. aureus* identification (Se: 80.0%; Sp: 98.8% and Acc: 98.0% in SCM samples and Se: 66.7%; Sp: 100% and Acc: 99.6% in PP cow samples), although the low prevalence of *Staph. epidermidis* and *Staph. saprophyticus* limit inferences about the performance of identifying these pathogens in Staph media. In conclusion, despite the limitation of the GP media for identification of *Staph. aureus*, GP, and Staph chromogenic media obtained satisfactory diagnostic performance results for the rapid identification of the main Gram-positive pathogens associated with SCM.

## Introduction

Mastitis is a disease that causes major losses in dairy production (1). The most prevalent form of the disease is subclinical mastitis (SCM) (2), which leads to decreased milk production, increased somatic cell count (SCC), and increased risk of clinical mastitis (CM) during lactation (3–5).

The occurrence of SCM in the dairy herd leads to direct as well as indirect economic losses, such as the decrease in milk production, which reduces the profitability of farms (6, 7). These losses are further worsened when SCM is caused by contagious agents, which can be transmitted between cows in the same herd (8), and, in this sense, controlling the spread of contagious SCM is essential for attaining higher profitability in dairy farms. In this situation, rapid identification of subclinical mastitis-causing pathogens is fundamental for rapid control measures.

The use of microbiological culture associated with biochemical tests is the standard method for identifying mastitis-causing agents. However, laboratory microbiological culture is not widely used in dairy herds across different countries (9). The main challenges for using laboratory microbiological culture are the logistical limitations and cost of shipping samples, along with the time of analysis to obtain the results, which can vary from 3 to 5 days (10). Alternatively, the on-farm microbiological culture has been used to rapidly identify clinical mastitis-causing pathogens, which allows greater agility in decision making for treatment, and, alternatively, it could also be used to choose preventive or control measures in the herd, such as segregation or culling (9). In similar ways, rapid and correct identification of SCM causing agents could assist in guiding measures to control mastitis pathogens in the herd, strategic treatments for intramammary infection (IMI), when treatment is viable, or selective dry cow therapy (10).

Chromogenic culture media are alternatives for rapid microbiological identification, as they make it possible to presumptively differentiate bacterial species and/or groups according to colony color, reducing the need for biochemical tests (11). These culture media outperform other conventional microbiological rapid methods (e.g., Minnesota Easy System Triplets; Mastitis SSGN Quad Culture Plates) concerning specificity (Sp), sensitivity (Se), and accuracy (Acc) (12), in addition to identifying contamination in the samples more efficiently (13), which minimizes the occurrence of false positives in the identification of agents.

The use of chromogenic media for detecting mastitis-causing pathogens has been described in Europe (13); North America (14) and South America (15). According to Ganda (2016), chromogenic media presented 100% Se and 99.8% Sp for the identification of *Staph. aureus* in CM milk samples. Therefore, the use of chromogenic culture media on the dairy farm routine could be an alternative for the rapid identification of SCM. However, no previous study evaluated the effectiveness of chromogenic media in milk samples from cows with SCM, and for the monitoring of cows in post-partum (PP); which is a period of the high risk of new IMI and the manifestation of IMI not cured during the dry period can manifest (16).

Thus, the present study hypothesizes that the chromogenic media Mastitis GP and Staph. (CHROMagar ™, Paris—France), which are selective for Gram-positive (GP) and *Staphylococcus* spp. (Staph), respectively, present high Acc, Se, and Sp for rapid identification of the main SCM causing agents during lactation and in the PP period compared to standard laboratory microbiological identification methods such as mass spectrometry.

## Materials and Methods

The present study was carried out under the ethical principles of animal experimentation and followed the rules established by the National Council for the Control of Animal Experimentation (CONCEA). The experimental protocol adopted was approved by the Ethics Committee on the Use of Animals (CEUA) of the Faculty of Veterinary Medicine and Zootechnics of the University of São Paulo under registration No. 4579250719.

### Herd Selection and Sample Collection

The milk samples were obtained from six dairy herds located in the states of São Paulo (*n* = 5) and Minas Gerais (*n* = 1), Brazil. Dairy herds were selected based on non-probabilistic convenience sampling (availability to participate in the study and proximity to the host institution, Milk Quality Research Laboratory—Qualileite, Pirassununga, SP). Herds had an average of 125 (± 60) lactating cows housed in compost-bedded pack barns, with average milk production of 27.5 (± 2.5) liters/cow/day. Also, all herds performed individual monthly SCC analysis of all lactating cows. Before the beginning of the study, milking personnel and/or those responsible for the udder health of each herd were trained for procedures of sampling collecting collection, and clinical mastitis diagnosis.

Five hundred and four lactating cows with SCM (SCC> 200,000 cells/mL) had composite milk samples (pool of the four mammary quarters) collected for microbiological identification of mastitis-causing pathogens. Additionally, 541 cows in the PP period (7 ± 3 days after calving) had composite milk samples (*n* = 226) or individual samples (*n* = 315) of mammary quarter collected for microbiological culture. In our study, cows with CM, or cows that present concomitant diseases other than mastitis and/or treated with antimicrobials (systemic or intramammary) in <14 days of sample collection, were excluded.

The procedures of milk sample collection for microbiological analysis were performed as described by NMC (17). Briefly, prior to milk collection, teat end was cleaned and disinfected with 70% iodized alcohol (70% alcohol + 2% iodine). The first milk strips were discarded and the milk was collected directly in a sterile tube, previously identified. The collection of milk samples from cows in PP was carried out by previously trained farm workers. Samples were stored at −20°C on the farm for a maximum period of 30 days until the microbiological culture analysis was carried out.

### Chromogenic Culture Media

Two selective chromogenic culture media, GP and Staph (CHROMagar ™, Paris, France), for identification of Gram-positive bacteria and *Staphylococcus* spp., respectively, were evaluated. The results of presumptive bacterial identification were visually performed based on the interpretation of the colony color characteristics, according to the manufacturer's recommendation.

**Figure 1:**
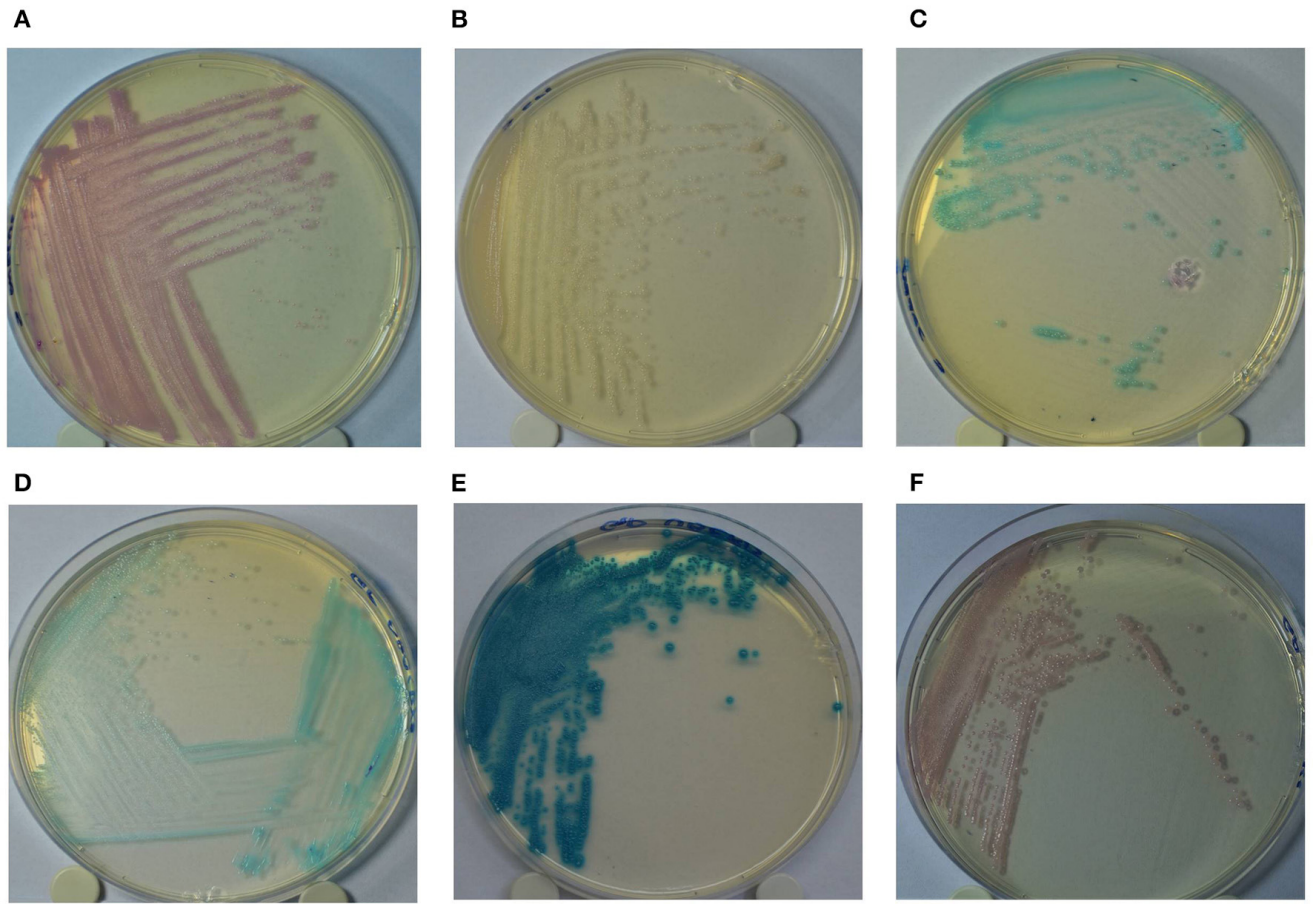
Colony color pattern of *Staphylococcus aureus* (**A**: pink); *Staphylococcus epidermidis* (**B**: pinkish/colorless); *Staphylococcus saprophyticus* (**C**: turquoise blue) grown in Staphylococcus chromogenic culture media **(A–C)**; and *Streptococcus agalactiae*/*Streptococcus dysgalactiae* (**D**: turquoise blue); *Streptococcus uberis/Enterococcus* spp. (**E**: dark/metallic blue) and *Staphylococcus aureus* (**F**: pink) grown in Mastitis GP culture media **(D–F)**.

The GP culture media identification results were interpreted according to the following colony colors: (a) turquoise blue—*Streptococcus agalactiae*/*dysgalactiae*; (b) dark blue/metallic blue—*Streptococcus uberis*/*Enterococcus* spp.; (c) pink/mauve—*Staphylococcus aureus* (Figure 1). Additionally, the identification results of Staph culture media were interpreted according to the following colony colorations: (a) pink—*Staph. aureus*; (b) colorless/pinky colony—*Staphylococcus epidermidis*; (c) turquoise blue—*Staphylococcus saprophyticus*. In both culture media, colonies showing other colors not described in the manufacturer's recommendations were considered as “other microorganisms.”

### Evaluation of Chromogenic Culture Media for Mastitis-Causing Agents Identification

For the microbiological identification of mastitis-causing pathogens, 0.01 mL of milk samples were inoculated, using a calibrated platinum loop, simultaneously in three culture media: a) blood agar supplemented with 5% bovine blood; b) GP chromogenic media and c) Staph chromogenic media. After inoculation, the plates were incubated at 37°C for 24 h, and then they were subjected to visual inspection to evaluate the microbiological growth. Samples that presented ≥ 3 microorganisms with different characteristics in composite milk samples, or individual mammary quarter, were considered contaminated (18). Besides, samples with the isolation of two microorganisms with different colony morphologies were considered “mixed culture.” In chromogenic media, colony colors were analyzed for microbiological identification, according to the manufacturer's recommendations (Figure 1). The evaluation of the bacterial colonies on the chromogenic culture media was carried out with a white background, for better differentiation of colony color. The microbial growth was recorded by a digital camera. In the GP chromogenic media, samples were considered positive for *Strep. uberis*/*Enterococcus* spp. or *Strep. agalactiae*/*dysgalactiae* when they presented a growth of 3 or more colonies with the color pattern defined for the species. For *Staph. aureus*, the growth of only 1 colony was considered the threshold for the identification of the agent. In the Staph culture media, the growth of ≥ 1 colony was sufficient for the identification of *Staph. aureus, Staph. epidermidis*, and *Staph. saprophyticus*. After the visual inspection of morphological characteristics, all the isolates that showed positive growth in blood agar and the chromogenic culture media were submitted to microbiological identification by Matrix Associated Laser Desorption-Ionization—Time of Flight (MALDI-TOF MS), as previously described (19). Throughout the experimental period, the evaluation of microbial growth in the culture media was carried out by the same evaluator, who had no prior knowledge of the results of identification by MALDI-TOF of the isolates.

### Diagnostic Performance

The visual identification of mastitis-causing agents by GP and Staph chromogenic media was compared to the gold standard method (MALDI-TOF MS) to estimate performance indicators. Samples that showed contamination in one of the two results (blood agar and/or chromogenic media) were excluded in the final analysis.

The performance indicators of Acc, Se, Sp, Positive Predictive Value (PPV), and Negative Predictive Value (NPV) of the chromogenic media were calculated based on the following results: True Positive (TP; when there was microbial growth and the result of visual microbiological identification of the chromogenic media was corresponding to that of the gold standard method), True Negative (TN; when there was no growth of any microorganism in the chromogenic culture media and blood agar), False Positive (FP; when there was a growth of any microorganism whose result was different between the microbiological identification of the gold standard method and the visual microbiological identification of the chromogenic media), False Negative (FN; when there was no growth of microorganisms in the chromogenic media and there was a growth of any microorganism in the gold standard method) (12).

The SENSPAC option of PROC FREQ; SAS version 9.4 (SAS INC., North Carolina, USA) was used to calculate Se, Sp, PPV, and NPV. Acc was also calculated by PROC FREQ, considering the TP and TN results. The results of Acc, Se, Sp, PPV, and NPV for the microbiological identification of chromogenic culture media were classified as low (results <60%), intermediate (between 60 and 80%), and high (> 80%) (20).

The results of Cohen's Kappa coefficient of agreement (k) were calculated by PROC FREQ of SAS version 9.4 (SAS INC., North Carolina, USA). Almost perfect agreement was considered when results of the coefficient between 0.81 and 1.00; substantial agreement (results from 0.61 to 0.80); moderate agreement (results from 0.41 to 0.60); fair agreement (results between 0.21 and 0.40), slight agreement (results between 0.00 and 0.20), and values ≤ 0.00 poor agreement (21).

## Results

A total of 504 composite milk samples from SCM cases were evaluated during the 02/01/2019–03/15/2019 periods. A total of 51 different species of microorganisms (that includes bacteria, yeast and algae) were isolated from the three culture media (GP; S and blood agar), considering that the microorganisms isolated in each media are not necessarily the same for the same sample. No contamination was observed in SCM samples evaluated.

A total of 541 milk samples (226 composite and 315 from mammary quarter) from post-partum dairy cows were evaluated during the 04/12/2019–07/25/2019 periods. A total of 36 different species of microorganisms (bacteria, yeast, and algae) were isolated considering the growth results for the three culture media evaluated. In 0.9% milk samples (*n* = 5; four composite and one of the mammary quarter) contamination was observed, and these milk samples were not evaluated in diagnostic performance analysis.

### Frequency of Mastitis Pathogens Identification

Based on blood agar growth, 61.3% (309/504) of the SCM milk samples and 30.5% (165/541) of PP milk samples had microbiological growth. Of these, mixed culture was observed in 3.8% (19/504) SCM and in 2.4% (13/541) PP milk samples. Gram-positive bacteria had higher isolation frequency (57.1%; 288/504 in SCM and 28.3%; 153/541 in PP) and non-aureus *Staphylococcus* (NAS) was the microorganism group that had the largest isolation frequency (25%; 126/504 in SCM and 22.6% 122/541 in PP). *Staph. chromogenes* was the most frequently isolated pathogen in both SCM (20.6%; 104/504) and PP (15.5%; 84/541; Table 1) milk samples.

**Table 1:** Frequency of mastitis-causing agents isolated from milk samples of lactating cows with subclinical mastitis and in post-partum period, identified using two chromogenic culture media (Gram-positive and Staphylococcus) and by MALDI-TOF MS.

	**SCM**^****a****^						**PP**^****b****^					
	**BA^**c**^**	**%**	**GP^**d**^**	**%**	**Staph^**e**^**	**%**	**BA**	**%**	**GP**	**%**	**Staph**	**%**
Total samples	504	100	504	100	504	100	541	100	541	100	541	100
Negative culture	195	38.6	255	50.6	339	67.3	371	68.6	464	85.8	506	93.5
Positive culture	309	61.3	249	49.4	165	32.7	165	30.5	72	13.3	30	5.5
Single morphology colonies	290	57.5	220	43.7	153	30.4	152	28.1	64	11.8	27	5.0
Mixed morphology^f^	19	3.8	29	5.8	12	2.4	13	2.4	8	1.5	3	0.6
Contamination^g^	0	0	0	0	0	0	5	0.9	5	0.9	5	0.9
Gram-positive bacteria	288	57.1	249	49.4	165	32.7	153	28.3	70	12.9	29	5.4
*Strep*.^h^ *agalactiae*	25	5	34	6.7	0	0	0	0	0	0	0	0
*Strep. dysgalactiae*	21	4.2	16	3.2	0	0	7	1.3	5	0.9	0	0
*Strep. uberis*	61	12.1	69	13.7	2	0.4	4	0.7	4	0.7	0	0
*Enterococcus* spp.	2	0.4	4	0.8	0	0	2	0.4	2	0.4	0	0
*Staph*.^i^ *aureus*	20	4	20	4	20	4	6	1.1	5	0.9	5	0.9
NAS^j^	126	25	102	20.2	124	24.6	122	22.6	48	8.9	19	3.5
*Staph. chromogenes*	104	20.6	83	16.5	98	19.4	84	15.5	39	7.2	11	2
*Staph. epidermidis*	1	0.2	0	0	2	0.4	4	0.7	2	0.4	1	0.2
*Staph. saprophyticus*	1	0.2	0	0	0	0	0	0	0	0	0	0
Other NAS	22	4.4	21	4.2	29	5.8	38	7	8	1.5	8	1.5
Other Gram-positive	48	9.5	23	4.6	26	5.2	17	3.1	6	1.1	6	1.1
Gram-negative bacteria	17	3.4	0	0	0	0	11	2	0	0	0	0
Other pathogens	5	1	2	0.4	0	0	1	0.2	1	0.2	0	0

a *subclinical mastitis samples;*

b *post-partum samples;*

c *blood agar;*

d *mastitis GP (CHROMagar^TM^. Paris—France);*

e *Staphylococcus (CHROMagar™. Paris—France);*

f *plates containing colonies with two different morphologies;*

g *colonies with more than two different morphologies;*

h *Streptococcus spp.;*

i *Staphylococcus spp.;*

j *Non-aureus Staphylococcus*.

In GP culture media, 49.4% (249/504) of SCM and 13.3% (72/541) of PP milk samples had microbiological growth. Mixed culture was observed in 5.8% (29/504) of SCM and 1.5% (8/541) of PP milk samples. Also, 4.8% (24/504) of SCM and 0.6% (3/541) of PP milk samples had microbiological isolation in GP chromogenic media and had no growth in blood agar. *Staph. chromogenes* were the most frequent pathogen isolated in GP media (16.5%; 83/504 in SCM and 7.2%; 39/541 in PP milk samples; Table 1).

In Staph chromogenic media, 32.74% (165/504) of SCM and 5.5% (30/541) of PP milk samples had microbiological isolation. Of these, mixed culture was observed in 2.4% (12/504) of SCM and 0.6% (3/541) of PP milk samples. Also, 5.6% (28/504) of SCM and 0.7% (4/541) of PP milk samples had no growth in blood agar and had microbiological isolate in Staph chromogenic media. *Staph. chromogenes* was the most isolated pathogen (19.4%; 98/504 in SCM and 2.0%; 11/541 in PP milk samples), followed by *Staph. aureus* (4.0%; 20/504 in SCM and 0.9%; 5/541 in PP). *Strep. uberis* was isolated in Staph media in 0.4% (2/504) of SCM milk samples. Colonies of *Strep. uberis* identified in the Staph media and the respective milk sample were re-inoculated in Staph media plates and no microbiological isolation was observed.

### Diagnostic Performance of Chromogenic Culture Media

Accuracy of GP chromogenic media identification varied according to the pathogen group from 92.3% (*Strep. uberis*/*Enterococcus* spp.) to 95.6% (*Strep. agalactiae*/*dysgalactiae*) for SCM milk samples, and from 98.9% (*Staph. aureus*) to 99.3% (*Strep. uberis*/*Enterococcus* spp.) for PP milk samples. Additionally, Se of GP media varied according to the group of pathogens from 25.0% (*Staph. aureus*) to 90.5% (*Strep. uberis*/*Enterococcus* spp.) in SCM milk samples, and from 50% (*Staph. aureus*) to 100% (*Strep. agalactiae*/*dysgalactiae*) for PP milk samples. Gram-positive culture media Sp for SCM ranged from 92.5% (*Strep. uberis*/*Enterococcus* spp.) to 98.7% (*Staph. aureus*), and in PP milk samples ranged from 99.1% (*Strep. agalactiae*/*dysgalactiae*) to 99.6% (*Strep. uberis*/*Enterococcus* spp.).

Positive predictive value results of GP media identification varied according to the pathogen group from 38.5% (*Staph. aureus*) to 70.4% (*Strep. agalactiae*/*dysgalactiae*) in SCM milk samples and 50.0% (*Staph. aureus*) to 71.4% (*Strep. uberis*/*Enterococcus* spp.) in PP milk samples. Also, NPV results range from 97.0% (*Staph. aureus*) to 98.9% (*Strep. agalactiae*/*dysgalactiae*) in SCM milk samples, and range from 99.4% (*Staph. aureus*) to 100% (*Strep. agalactiae*/*dysgalactiae*) in PP milk samples.

Kappa coefficient of agreement values varied according to the pathogen group from 0.27 (*Staph. aureus*) to 0.76 (*Strep. agalactiae*/*dysgalactiae*) in SCM milk samples and range from 0.45 (*Staph. aureus*) to 0.72 (*Strep. uberis*/*Enterococcus* spp.) in PP milk samples. Indicators of diagnostic performance for negative results of SCM in GP chromogenic media were: Acc = 82.1%, Se = 88.7%, Sp = 77.3%, PPV = 74.1%, NPV = 90.4% and *k* = 0.60. Similarly, for PP negative milk samples, Acc was 83.4%, Se = 99.2%, Sp = 44.5%, PPV = 81.5%, NPV = 95.8%, and *k* = 0.50.

Considering the low isolation frequency of *Staph. epidermidis* (*n* = 1 in SCM and *n* = 4 in PP milk samples) and *Staph. saprophyticus* (*n* = 1 in SCM and *n* = 0 in PP milk samples), it was not possible to evaluate the diagnostic performance of Staph chromogenic media for microbiological identification of these pathogens. However, *Staph. aureus* identification by Staph media in SCM milk samples had Acc = 98.0%, Se = 80.0%; Sp = 98.8%; PPV = 72.7% and NPV = 99.2%. In addition, for *Staph. aureus* identification in PP milk samples, Staph media had results of Acc = 99.6%; Se = 66.7%; Sp = 100%; PPV = 100%, NPV = 99.6%, and *k* was 0.71 for SCM and 0.80 for PP milk samples (Table 2). Indicators of diagnostic performance for negative results of SCM in Staph chromogenic media were: Acc = 86.3%, Se = 91.4%, Sp = 77.0%, PPV = 87.9%; NPV = 83%, and *k* = 0.53 (Table 3). Similarly, for PP milk samples that had negative results the Acc was 80.4%, Se was 99.0%, Sp was 20.5%, PPV was 80.0%, NPV was 86.7%, and *k* was 0.29.

**Table 2:** Indicators of diagnostic performance of the Gram-positive chromogenic media in milk samples from cows with SCM (*n* = 504) and cows in PP (*n* = 536).

**Indicator**		**Gram-positive chromogenic media**^****a****^			
	**Negative**	***Strep. uberis/Ent*.^**b**^**	***Strep. aga*/dys^**c**^**	***Staph. aureus*^**d**^**
N^e^				
SCM^f^	249	63	46	20
PP^g^	433	6	7	6
Acc%^h^				
SCM	82.1 (78.8–85.5)	92.3 (89.9–94.6)	95.6 (93.9–97.4)	95.4 (93.6–97.3)
PP	98 (96.8–99.2)	99.3 (98.5–100)	99.1 (98.3–99.9)	98.9 (98–99.8)
Se%^i^				
SCM	88.7 (84.5–93)	90.5 (83.2–97.7)	89.1 (80.1–98.1)	25 (6–44)
PP	99.2 (98.3–100)	71.4 (38–100)	100 (100–100)	50 (10–90)
Sp%^j^				
SCM	77.3 (72.5–82.1)	92.5 (90–95)	96.3 (94.6–98)	98.3 (97.2–99.5)
PP	44.5 (36.7–52.3)	99.6 (99.1–100)	99 (98.2–99.9)	99.4 (98.8–100)
PPV%^k^				
SCM	74.1 (68.7–79.5)	63.3 (53.4–73.3)	70.7 (59–82.4)	38.5 (12–64.9)
PP	81.4 (77.9–85)	71.4 (38–100)	58.3 (30.4–86.2)	50 (10–90)
NPV%^l^				
SCM	90.4 (86.7–94)	98.5 (97.4–99.7)	98.9 (97.9–99.9)	96.9 (95.4–98.5)
PP	95.8 (91.2–100)	99.6 (91.1–100)	100 (100–100)	99.4 (98.8–100)
*k* (IC 95%)^m^				
SCM	0.60 (0.53–0.67)	0.71 (0.61–0.79)	0.76 (0.61–0.79)	0.30 (0.09–0.5)
PP	0.50 (0.42–0.59)	0.72 (0.42–1)	0.69 (0.46–0.93)	0.49 (0.14–0.84)
*k*—*P* Value^n^				
SCM	<0.0001	<0.0001	0.01	0.04
PP	<0.0001	0.56	0.01	1

a *gram-positive selective chromogenic media- Mastitis GP (CHROMagar^TM^. Paris—France);*

b *Streptococcus uberis/Enterococcus spp.;*

c *Streptococcus agalactiae/dysgalactiae;*

d *Staphylococcus aureus;*

e *isolation frequency;*

f *subclinical mastitis samples;*

g *post-partum samples;*

h *accuracy;*

i *sensitivity;*

j *specificity;*

k *positive predictive value;*

l *negative predictive value;*

m *Cohen's Kappa concordance test;*

n *Kappa Coefficient P-value*.

**Table 3:** Indicators of diagnostic performance in milk samples from cows with SCM (*n* = 504) and cows in PP (*n* = 536).

**Indicator**		***Staphylococcus*** **chromogenic media**^****a****^			
	**Negative**	***Staph. aureus*^**b**^**	***Staph. epi*.^**c**^**	***Staph. sapro*.^**d**^**
N^e^				
SCM^f^	338	20	1	1
PP^g^	493	6	4	0
Acc%^h^				
SCM	86.3 (83.3–89.3)	98 (96.8–99.2)	98.6 (97.6–99.6)	96.2 (94.6–97.9)
PP	80.4 (77–83.8)	99.6 (99.1–100)	99.3 (98.5–100)	98.9 (9 8–99.8)
Se%^i^				
SCM	91.4 (88.4–94.5)	80 (62.5–97.5)	0 (.)	0 (.)
PP	99 (98.1–100)	66.7 (29–100)	25 (0–67.4)	.
Sp%^j^				
SCM	77 (70.8–83.2)	98.8 (97.8–99.8)	98.8 (.)	96.4 (.)
PP	20.5 (13.5–27.5)	100 (100–100)	99.8 (99.4–100)	98.9 (.)
PPV%^k^				
SCM	87.9 (84.4–91.4)	72.7 (54.1–91.3)	0 (.)	0 (.)
PP	80 (76.6–83.5)	100 (100–100)	50 (0–100)	0 (.)
NPV%^l^				
SCM	83 (77.3–88.8)	99.2 (98.4–100)	99.8 (.)	99.8 (.)
PP	86.7 (74.5–98.8)	99.6 (99.1–100)	99.4 (98.8–100)	100 (.)
*k* (IC 95%)^m^				
SCM	0.53 (0.44–0.61)	0.73 (0.56–0.89)	(−0.003) [(−0.008)−0.002]	(−0.004) [(−0.010)−0.003]
PP	0.29 (0.19–0.39)	0.80 (0.52–1.00)	0.33 [(−0.16)−0.82]	0
*k*—*P* Value^n^				
SCM	<0.0001	0.52	0.17	0.006
PP	<0.0001	0.15	0.31	.

a *selective chromogenic media to Staphylococcus spp.—Staph. (CHROMagar™. Paris—França);*

b *Staphylococcus aureus;*

c *Staphylococcus epidermidis;*

d *Staphylococcus saprophyticus;*

e *isolation frequency;*

f *subclinical mastitis samples;*

g *post-partum samples;*

h *accuracy;*

i *sensitivity;*

j *specificity;*

k *positive predictive value;*

l *negative predictive value;*

m *Cohen's Kappa concordance test.;*

n *Kappa Coefficient P-value*.

## Discussion

Chromogenic culture media may be used as alternatives for rapid microbiological identification, considering the potential to differentiate species and/or groups of microorganisms (11). This study evaluated the ability of GP and Staph chromogenic culture media for rapid identification of mastitis-causing pathogens in milk from SCM and cows in PP.

In chromogenic media GP, it was observed high Se and Sp for identification of *Strep. uberis*/*Enterococcus* spp. and *Strep. agalactiae/dysgalactiae* groups, both in SCM and PP samples. Se and Sp values obtained for SCM samples (Se = 89.1%; Sp = 96.3% for *Strep. agalactiae/dysgalactiae* and Se = 90.5%; Sp = 92.5% for *Strep. uberis*) were similar to those obtained for the identification of *Streptococcus* spp. from the Minnesota Tri-Plate triplet (92.6 and 89.5%, respectively) (9); and also, for the results obtained by the Accumast chromogenic media (Se: 90% and Sp: 93%, respectively) (14). However, direct comparisons between our results and previous studies are limited because Ganda et al. (14) and MacCarron et al. (9) did not differ between *Strep. uberis* and *Strep. agalactiae/dysgalactiae*, grouping them into *Streptococcus* spp. This differentiation capacity observed in the chromogenic media GP enables farms to specifically identify the group and/or pathogen species and to adopt specific prophylactic control measures according to the species of *Streptococcus* spp. isolated, since these agents have distinct pathogenicity and transmission profile. The high Se and SP in PP samples (Se = 100%; Sp = 99% for *Strep. agalactiae/dysgalactiae* and Se = 71.4%; Sp = 99.6% for *Strep. uberis*) indicate that it is possible to obtain identification results reliable for these pathogens from PP samples.

The positive predictive values were classified as low/moderate for the *Strep. agalactiae/dysgalactiae* and *Strep. uberis/Enterococcus* spp. groups, both in SCM samples (PPV = 70.7% and PPV = 63.3; respectively) and for PP samples (PPV = 58.3 and PPV = 71.4; respectively). These results were inferior to those obtained by Ganda et al. (14) (Accumast) and MacCarron et al. (9) (Minnesota plate) but they were close to those reported by both the Minnesota Easy Culture System Bi-Plate and Tri-Plate (20). However, if we take into account the isolates that were grown in the GP media that were not isolated on blood agar, the diagnostic performance would be higher, especially in the samples of SCM (PPV = 89.7% for *Strep. agalactiae/dysgalactiae* and PPV = 80% for *Strep. uberis/Enterococcus* spp.) which would match the results obtained by Accumast and Minnesota plate.

The NPV result of the groups of *Strep. agalactiae/dysgalactiae* and *Strep. uberis/Enterococcus* spp. was considered high for both SCM samples (NPV = 98.9% and NPV = 98.5%; respectively) and for PP samples (NPV = 100% and NPV = 99.6). The results obtained were superior to those of the Minnesota Easy Culture System biplane for *Streptococcus* spp., and similar to those obtained by the Minnesota Easy Culture System Tri-Plate (20) and by the Accumast method (14). The high NPV values indicate the assertiveness of negative results in GP media when there is no isolation of agents from the *Strep. agalactiae/dysgalactiae* and *Strep. uberis/Enterococcus* spp. groups.

The Se values of the chromogenic media GP for presumptive identification of *Staph. aureus* was lower concerning the other groups of pathogens for samples of SCM (Se = 25%) and PP samples (Se = 50%). This low Se can be attributed to an inconsistency in the color pattern generated for the diversity of *Staph. aureus* isolates that were evaluated in this study. Different shades of pink were observed, not necessarily consistent with the characteristic color associated with *Staph. aureus* identification according to the manufacturer's recommendations, which caused misidentification and high numbers of FN results. Due to the high amount of FN results, both in SCM samples (FN = 75%; 15/20) and in PP samples (FN = 50% 3/6), PPV was also compromised (SCM = 38.5% and PP = 50%, respectively). Similar results for Se and PPV were obtained for *Staph. aureus* in SCM samples by the rapid microbiological identification method VetoRapid kit (22) and CM by the SSGN and SSGNC quadrates (12). Contrary to our results for SCM samples, 3M Petrifilm system (9), and, in CM Accumast (14) and Minnesota Easy Culture System II (20), presented high Se and PPV results for *Staph. aureus* identification. In our study, SCM composite milk samples were used, which may result in a lower bacterial count necessary for the isolation of microorganisms in comparison to CM samples. Thus, it is possible to have a lower Se in the isolation of microorganisms in composite samples concerning CM samples per mammary quarter (23).

For the chromogenic media Staph, the results of Se and Sp for *Staph. aureus* was high in SCM samples (Se = 80%; Sp = 98.8%) and moderate to high for PP samples (Se = 66.7%; Sp = 100%; respectively). The PPV result was intermediate for SCM samples (PPV = 72.7) and the NPV result was high (NPV = 99.2). However, PPV would be higher (PPV = 90.9%) if we included samples with the isolation of *Staph. aureus* in Staph culture media (correctly identified by the chromogenic media), but without growth on blood agar were considered TP. These results are similar to those obtained by Accumast (14) and Minnesota Easy Culture System II (20). The ability to rapidly identify *Staph. aureus* infected cows are essential for the control of SCM in problem-herds, considering the potential of contagious transmission and low cure risk (24). The results of *Staph. aureus* identification by chromogenic media Staph can assist in decision making regarding the segregation and/or culling of *Staph. aureus* infected cows. Besides, the Staph chromogenic media makes it possible to differentiate between two other species of *Staphylococcus* spp. (*Staph. saprophyticus* and *Staph. epidermidis*), which has not been described in Minnesota Easy Culture System II and Accumast. However, due to the low isolation frequency of *Staph. saprophyticus* and *Staph. epidermidis*, it was not possible to determine the diagnostic performance in these species.

The agreement between the GP chromogenic media and the gold standard method, as measured by the *k* coefficient, was substantial for the *Strep. agalactiae/dysgalactiae* (*k* = 0.76) and *Strep uberis/Enterococcus* spp. (*k* = 0.70) groups for SCM samples. In PP samples, we also observed substantial agreement for the groups of pathogens mentioned above (*k* = 0.72 for *Strep. uberis/Enterococcus* spp. and *k* = 0.69 for *Strep. agalactiae/dysgalactiae*). The agreement observed in the present study was higher than that obtained for *Streptococcus* spp. by the bi-plate (*k* < 0.6) and similar to the tri-plate (*k* between 0.73 and 0.85) Minnesota Easy Culture System (20), however, it was lower than that obtained by Accumast (*k* = 0.91) (14). The ability to discern between *Strep. uberis/Enterococcus* spp. and *Strep. agalactiae/dysgalactiae* groups can help control SCM, making possible, for example, the segregation and strategic treatment of cows presumptively positive for *Strep. agalactiae*, which is the only SCM causing agent which treatment during lactation is indicated (25).

In SCM samples, the *k* result obtained indicates that GP chromogenic media had a fair agreement for the rapid identification of *Staph. aureus* (k = 0.30), which corroborates the unsatisfactory results of Se (25.0%), and PPV (38.5%). For PP samples, the agreement was moderate (*k* = 0.49). The agreement results were lower than those obtained by Accumast (*k* = 0.93) (14) and by the Minnesota Easy Culture System Bi-Plate and Tri-Plate (*k* = 0.62 and k = 49; respectively) (20) but were similar to those obtained for the CHROMagar Mastitis chromogenic tri-plate (*k* = 0.33) (13). The low *k* coefficient for *Staph. aureus* identification indicates a limitation of the GP media.

Staph chromogenic media had a substantial agreement for the rapid identification of *Staph. aureus* for both SCM samples (*k* = 0.73) and PP samples (*k* = 0.79). Our results were slightly lower than the almost perfect agreement obtained by Accumast (*k* = 0.93) (14) in CM samples, which may be associated with a higher concentration of pathogens present in CM samples compared to SCM samples. However, the *k* coefficient of the Staph media was higher than that obtained by the Minnesota Easy Culture System Bi-Plate and Tri-Plate (both *k* < 0.7) (20), CHROMagar Mastitis, Hardy Diagnostics Mastitis Tri-Plate, and VetoRapid chromogenic triplets (*k* = 0.33 and *k* = 0.40, respectively) (13), indicating high agreement between the reference method and the presumptive identification of *Staph. aureus* by Staph culture media.

In the case of samples with no growth in the GP chromogenic media, high Se (88.7%) and moderate Sp (77.3%) results were obtained in SCM samples, indicating the assertiveness of the media for negative results. In the PP samples, GP chromogenic media obtained high Se for negative results (99.2%) but presented low Sp (44.5%). The low Sp may be associated with the high amount of FP (*n* = 86). The results obtained for the GP chromogenic media were superior to those described by Ferreira et al. (12) for the Accumast, Minnesota Easy System, SSGN, and SSGNC media, which may be partially associated with the selective capacity of the media.

The Se of samples without growth in the Staph chromogenic media was also high in SCM (Se = 91.4%), while the Sp was moderate (Sp = 77%). As observed in the GP chromogenic media, for PP samples, Sp was low (Sp = 20.5%) and Se was high (Se = 99%). The results indicate assertiveness in the diagnosis of negative samples for microbiological isolation, but little Sp in this identification, which again may be partially associated with the occurrence of high numbers of FP results, especially in PP samples (*n* = 101). Also, two samples of SCM had isolation of *Strep. uberis*, a pathogen that should have growth inhibited in the Staph chromogenic media, which is selective for *Staphylococcus* spp. However, in both cases, when these colonies were transferred to a new plate of the Staph chromogenic media, there was no microbial isolation, and the same occurred when a new inoculation of the milk sample was performed.

It should be noted that our study has some limitations. Visual inspection of the colonies and the presumptive identification of the pathogens in chromogenic media were performed by only one researcher and at the laboratory conditions, which makes it difficult to extrapolate the result to farm conditions. However, to minimize bias, the reader was blinded to the results of identification on the gold standard.

The main limitation of this study was the use of MALDI-TOF MS as the only reference method for comparing the results of bacterial identification in two chromogenic media. The identification of isolates is based on the manufacturer's spectral database of the specific species or strains (26). Since the Biotyper library is mainly based on human pathogens strains, the identification of some mastitis isolates is not possible. However, this limitation can be solved by adding mass spectra of strains from mastitis-causing pathogens in the local library (27). On the other hand, isolates correctly identified by chromogenic media (agreement between visual identification on the chromogenic media and by MALDI-TOF MS) were considered FP, when no bacterial growth was observed in blood agar. For example, a total of 31 isolates had correct identification in the GP media (29 from SCM and 2 from PP) and six isolates in the Staph media (*n* = 6 samples from SCM), which were considered FP results. These results may suggest that the selectivity of the chromogenic media could reduce the competition between microorganisms, thus allowing the growth of non-isolated microorganisms in blood agar (11).

In general, regardless of the type of sample (SCM or PP), with the only exception of *Staph. aureus* in GP, both chromogenic culture media tested showed satisfactory performances for the rapid identification of the pathogens they were designed to identify. The GP chromogenic media was efficient for the identification of the *Strep. agalactiae/dysgalactiae* and *Strep uberis/Enterococcus* spp. groups, enabling the differentiation between *Strep. uberis* and *Strep. agalactiae/dysgalactiae* without the use of additional biochemical tests (e.g., Christie, Atkins, Munch-Petersen test). The ability of GP chromogenic culture media to differentiate between the groups *Strep. uberis*/*Enterococcus* spp. from *Strep. agalactiae*/*dysgalactiae* would be helpful for immediate SCM control, such as the segregation of suspected positive cows for *Strep. agalactiae*. Additionally, the identification of *Strep. agalactiae*/*dysgalactiae* positive cows using the GP media is an indication of the need to screen the whole herd for identification of *Strep. agalactiae* cows based on laboratory microbiological identification.

In this study, GP media presented limitations in the identification of *Staph. aureus*, which is possibly related to the inconsistency of the color pattern associated with the identification of this pathogen. The performance of the Staph chromogenic media was generally satisfactory for *Staph. aureus*, the main pathogen to which the media proposed to identify, what indicates the potential of the Staph media to be used in on-farm culture systems for specific identification of *Staph. aureus*. The identification of *Staph. aureus* positive cows would be helpful for control measures, such as the segregation and/or culling of infected cows.

Finally, the tested chromogenic media obtained diagnostic performance similar to other methods of rapid microbiological identification used in on-farm culture systems, with the advantage of allowing between *Strep. uberis* and *Strep. agalactiae/dysgalactiae* in GP media, as well as it possible to differentiate between *Staph. aureus*; *Staph. epidermidis* and *Staph. saprophyticus*, when using Staph media.

## Conclusion

The results of diagnostic performance of GP and Staph chromogenic media were satisfactory in identifying the main pathogens associated with mastitis in milk samples from cows in PP and SCM samples during lactation, except for *Staph. aureus* identification using GP media. The possibility of rapid microbiological identification (from 18 to 24 h), the high values obtained for the diagnostic performance in most groups of pathogens, and the ability to differentiate between some species of *Staphylococcus* spp. and *Streptococcus* spp. genus suggests that GP and Staph media are adequate alternatives for using rapid identification of Gram-positive pathogens causing SCM.

## Data Availability Statement

The raw data supporting the conclusions of this article will be made available by the authors, without undue reservation.

## Ethics Statement

The animal study was reviewed and approved by National Council for the Control of Animal Experimentation (CONCEA) Ethics Committee on the Use of Animals (CEUA) of the Faculty of Veterinary Medicine and Zootechnics of the University of São Paulo. Written informed consent was obtained from the owners for the participation of their animals in this study.

## Author Contributions

BGa and MS took part in designing the experiment. BGa, CF, GF, BGr, and MS performed the experiment, data analysis, and wrote the paper. All authors participated in reading, provided a critical review, and approved the final manuscript.

## Conflict of Interest

The authors declare that the research was conducted in the absence of any commercial or financial relationships that could be construed as a potential conflict of interest.
